# Natural Genetic Variation Influences Protein Abundances in *C*. *elegans* Developmental Signalling Pathways

**DOI:** 10.1371/journal.pone.0149418

**Published:** 2016-03-17

**Authors:** Kapil Dev Singh, Bernd Roschitzki, L. Basten Snoek, Jonas Grossmann, Xue Zheng, Mark Elvin, Polina Kamkina, Sabine P. Schrimpf, Gino B. Poulin, Jan E. Kammenga, Michael O. Hengartner

**Affiliations:** 1 Institute of Molecular Life Sciences, University of Zurich, Zurich, Switzerland; 2 Functional Genomics Center Zurich, University of Zurich and ETH Zurich, Zurich, Switzerland; 3 Laboratory of Nematology, Wageningen University, Wageningen, The Netherlands; 4 Faculty of Life Sciences, The University of Manchester, Manchester, United Kingdom; Fred Hutchinson Cancer Research Center, UNITED STATES

## Abstract

Complex traits, including common disease-related traits, are affected by many different genes that function in multiple pathways and networks. The apoptosis, MAPK, Notch, and Wnt signalling pathways play important roles in development and disease progression. At the moment we have a poor understanding of how allelic variation affects gene expression in these pathways at the level of translation. Here we report the effect of natural genetic variation on transcript and protein abundance involved in developmental signalling pathways in *Caenorhabditis elegans*. We used selected reaction monitoring to analyse proteins from the abovementioned four pathways in a set of recombinant inbred lines (RILs) generated from the wild-type strains N2 (Bristol) and CB4856 (Hawaii) to enable quantitative trait locus (QTL) mapping. About half of the cases from the 44 genes tested showed a statistically significant change in protein abundance between various strains, most of these were however very weak (below 1.3-fold change). We detected a distant QTL on the left arm of chromosome II that affected protein abundance of the phosphatidylserine receptor protein PSR-1, and two separate QTLs that influenced embryonic and ionizing radiation-induced apoptosis on chromosome IV. Our results demonstrate that natural variation in *C*. *elegans* is sufficient to cause significant changes in signalling pathways both at the gene expression (transcript and protein abundance) and phenotypic levels.

## Introduction

Most complex traits, including many common diseases such as cancer, neurodegenerative, and autoimmune diseases are affected by multiple genes. There is overwhelming evidence that individuals with different genotypes have a different susceptibility to various complex diseases. For instance, the genetic background influences onset and progression of cancer in mice and humans [1–3] and also plays an important role in other complex diseases such as renal failure [4], autoimmune diseases [5], and retinal degeneration [6]. Studying natural genetic variation is crucial for understanding the effect of allelic variation of gene expression and for determining the genetic basis of complex traits. However, the genetic mechanisms underlying these effects are often poorly understood.

To unravel how the genetic background affects signalling pathways that contribute to complex diseases, we used the model organism *Caenorhabditis elegans* [7]. Specifically, we selected genes involved in apoptosis [8], as well as genes involved in three pathways (MAPK [9], Notch [10], and Wnt [11]) that control vulva development, an important phenotypic readout in *C*. *elegans* for these pathways [12,13]. All four signalling pathways are evolutionarily conserved and many human homologs of these *C*. *elegans* signalling proteins [14] have been linked to various cancers, as well as neurodegenerative, cardiovascular, and other diseases [15–19].

Signalling pathways in *C*. *elegans* are usually studied in the canonical wild-type N2 (Bristol [20]) background through the screening for and characterization of induced mutations, which often lead to complete loss of gene function and show distinctive phenotypic defects. By contrast, natural variation usually causes more subtle phenotypic changes, as natural selection rapidly selects against mutations with strong negative impact.

To explore the effect of natural variation, we selected two highly divergent wild-type strains [21,22], N2 and CB4856 (Hawaii [23]), and a set of recombinant inbred lines (RILs) generated from them [24–27]. Previous transcriptome analysis of N2, CB4856, and RILs from this set showed significant heritable variation in gene expression at the transcript level, which could be mapped to several expression QTLs (eQTLs) [24,28–31], but very little is known about heritable variation at the protein level. Here we quantified the abundances of selected proteins from four cancer signalling pathways (apoptosis, MAPK, Notch, and Wnt) using selected reaction monitoring (SRM), a mass spectrometric technique for targeted quantitative proteomics that is characterized by its high specificity, sensitivity, and wide dynamic range [32,33]. We also measured apoptosis levels in both parental strains and RILs. We found that natural variation in *C*. *elegans* causes significant changes in signalling pathways both at the gene expression (transcript and protein abundance) and at the phenotypic levels.

## Results and Discussion

We selected 156 proteins (S1 Table) that have been implicated in one of four signalling pathways in *C*. *elegans*: apoptosis (62 proteins), MAPK (59 proteins), Notch (18 proteins), and Wnt (17 proteins). These 156 proteins span over three orders of magnitude in abundance [34], with approximately half of them being in the range of 1–20 ppm (S1A Fig and S1 Table), and show a similar abundance distribution as the overall *C*. *elegans* proteome (S1B Fig).

### SRM measurements

**Fig 1:**
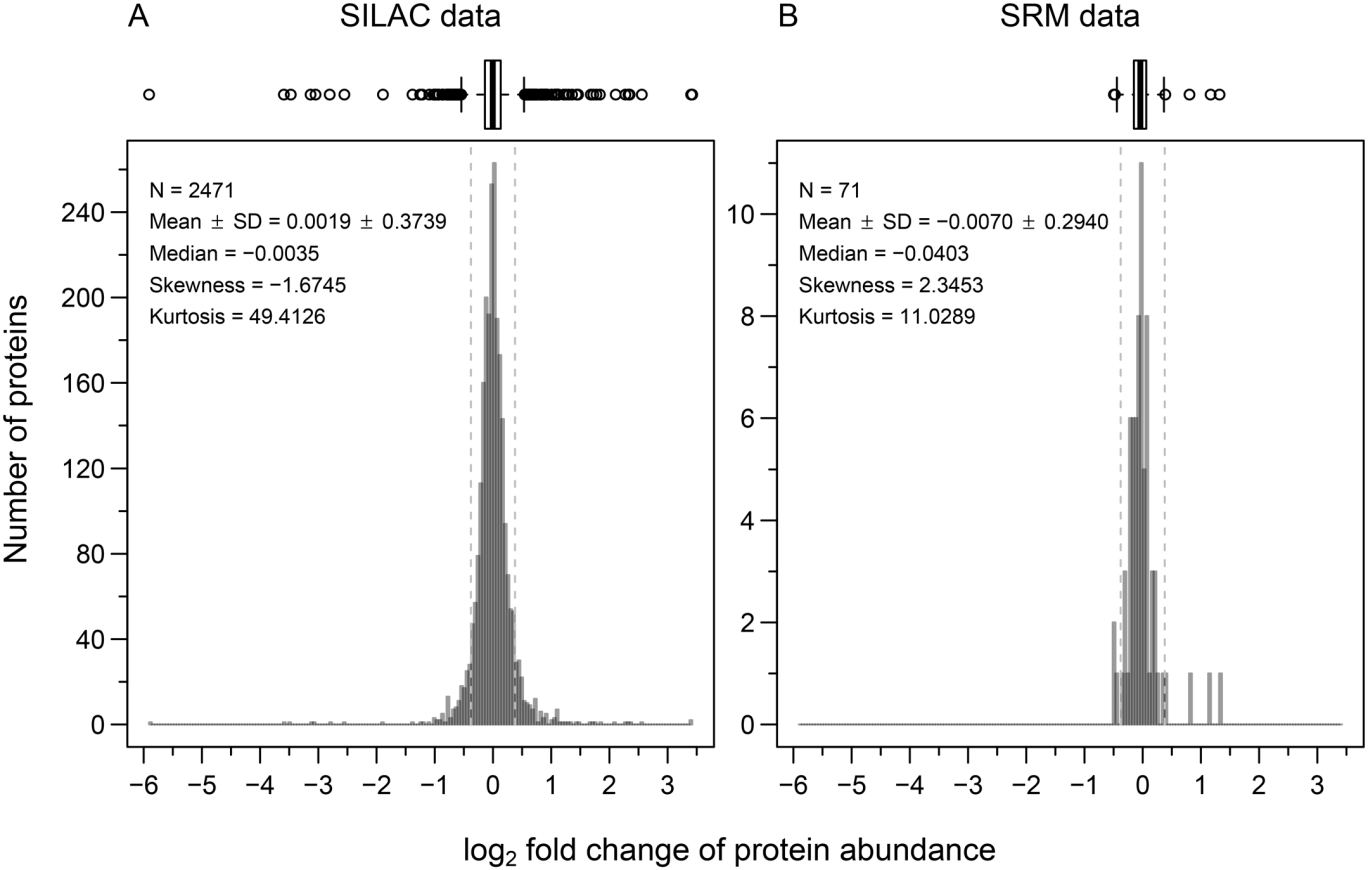
Signalling pathway proteins show a similar variation in abundance between N2 and CB4856 as a *C*. *elegans* shotgun proteome dataset. Histogram with Tukey-style box plot [36] on the top for protein abundances measured in CB4856 relative to N2. Vertical dashed lines represent the fold change cut-off of 1.3 (~ 0.38 on log_2_ scale). (A) The *C*. *elegans* shotgun proteome dataset was quantified using SILAC (data from [35]). (B) Signalling pathway proteins were quantified using SRM.

In a first step, we performed an experiment to find proteins that are differently abundant in the two parental strains N2 and CB4856. Out of 148 tested proteins (S2 Fig and S2 Table) we successfully measured the abundance of between 104 and 116 proteins in each sample; 71 proteins could be quantified in two biological replicates in both strains (S3 Fig). For most of these, the protein abundance difference was very small between the two strains (below our fold change cut-off, which we set at 1.3-fold, based on the variation in abundance that we measured between biological replicates of the same strain; S4 Fig) and also correlated poorly with the differences in transcript abundance levels (S5 Fig). The extent of conservation in protein abundance in the dataset of the four signalling pathways is very similar to the variation that can be observed at the level of the whole proteome using stable isotope labelling by amino acids in cell culture (SILAC) based shotgun mass spectrometry data from [35] (Fig 1 and S4 Fig). Taken together, these observations suggest that for most of the measured proteins, the small variation in protein abundance observed between CB4856 and N2 is not due to genetic variation between the two strains, but rather comes from measurement errors or from the innate variation that also exists between biological replicates of the same strain (S4 Fig).

**Fig 2:**
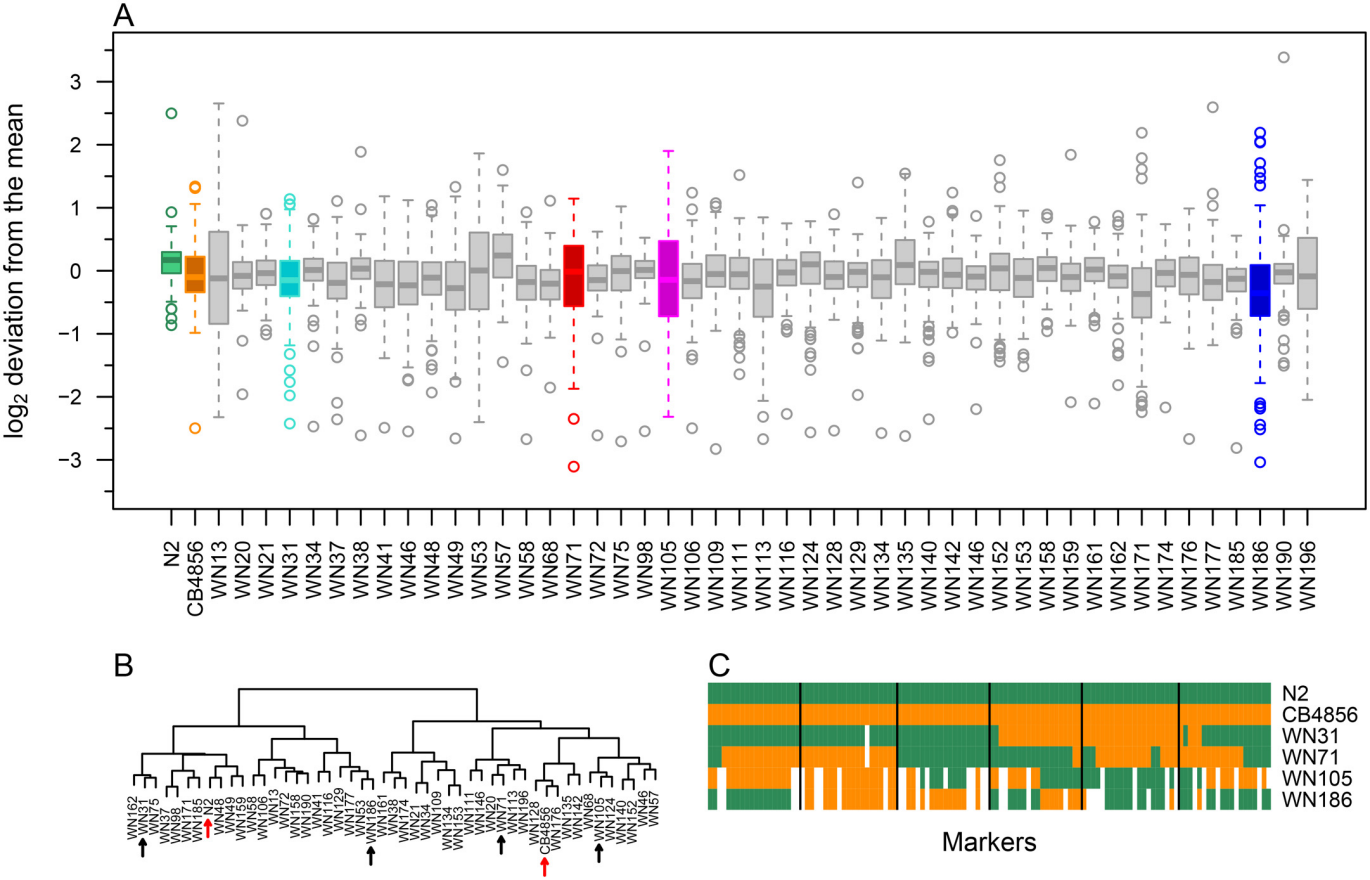
Many RILs have a higher transcript expression level variation than the parental strains. Gene expression at the transcript level was quantified in N2, CB4856, and 47 RILs by two-colour microarray analysis. (A) Tukey-style box plot representing log_2_ scaled deviations of the gene expression value from the mean across all samples. Four genetically different RILs (WN31, WN71, WN105, and WN186) that showed large variation in transcript abundance were selected for proteome quantification. (B) Hierarchical clustering of the 47 RILs and the two parental strains based on their genotype (see “Additional file 1” of [25] for details). Clustering was done with R functions “dist” and “hclust” from the package stats (version 3.2.2) using “euclidean” distance and “ward.D2” method. Four RILs (black arrows) were chosen from different clusters to ensure maximum genetic diversity; red arrows indicate parental strains. (C) Genotype of the four selected RILs and the two parental strains. Vertical lines separate chromosomes I to V and X from left to right.

Previous work on wild isolates has shown that wild-type strains often contain hidden variation in gene expression due to compensatory mutations within the genome [37,38]. Crossing of two wild types followed by the generation of RILs can lead to significant variation due to shuffling of the parental genotypes and the segregation of compensatory mutations [30,37,38]. To test this concept at the proteome level, we analysed RILs generated from the two wild types. As was previously reported [24,28,29], many RILs show a higher level of variation in transcript abundance than either parental strain (Fig 2A). We selected four RILs (WN31, WN71, WN105, and WN186) that showed large variation in transcript abundance for the 148 selected proteins compared with the parental strains (Fig 2A) and that also showed great genetic diversity between them (Fig 2B and 2C).

**Fig 3:**
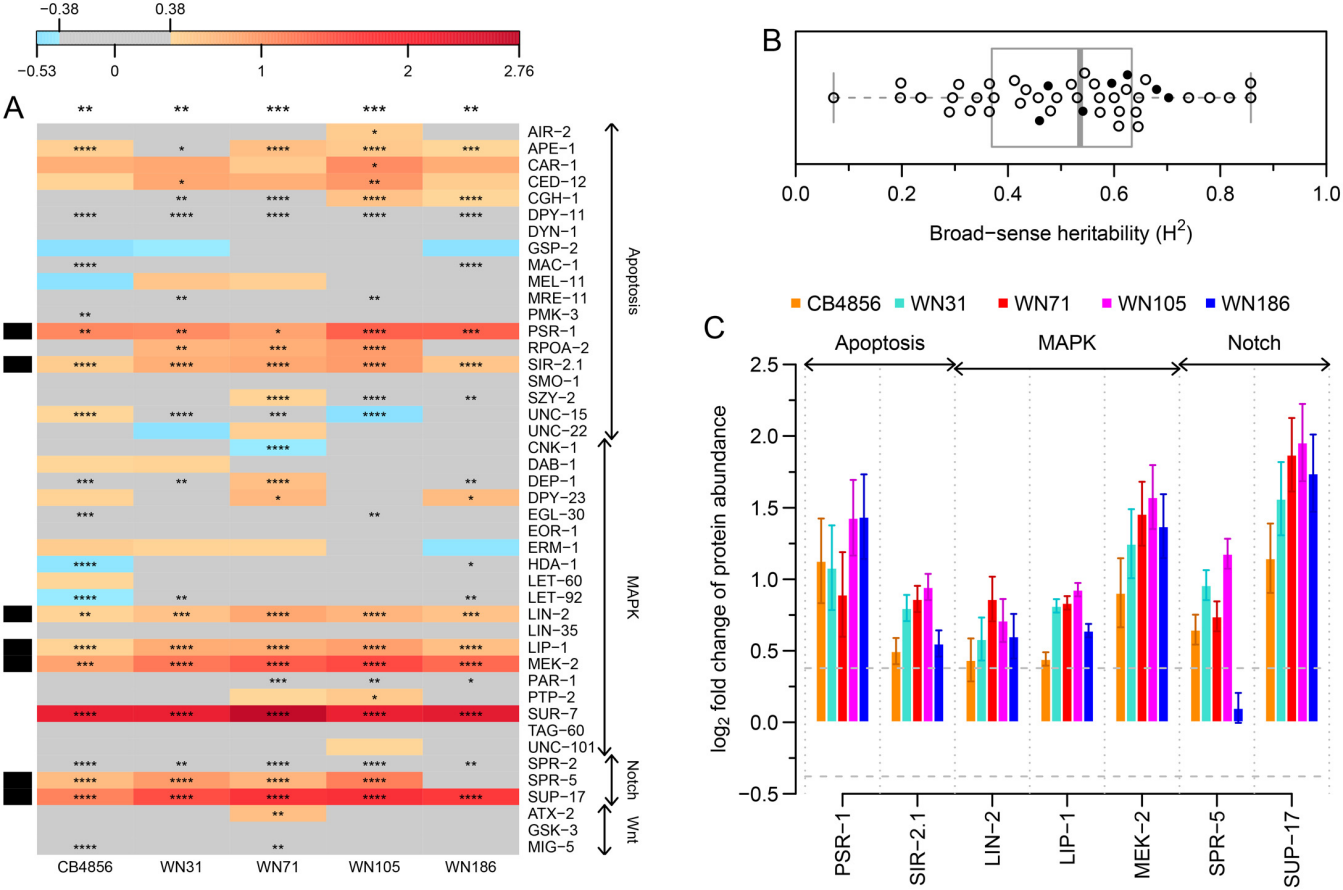
Signalling pathway proteins tend to be up-regulated in CB4856 and in RILs compared to N2. Protein abundance was quantified by SRM. Identification of the true peak group was performed using the mProphet software, followed by protein significance analysis using intensity-based linear mixed-effects model implemented in MSstats. (A) Heat map showing differential abundance of 44 proteins in CB4856 and four empirically selected RILs relative to N2. Blue and red shades represent log_2_ scaled fold changes, grey colour shows the fold change cut-off of 1.3 (~ 0.38 on log_2_ scale) and number of asterisks represent BH corrected *P*-values. Black bands on the left side indicate proteins selected for subsequent pQTL mapping. Number of asterisks on top of each column represent BH corrected *P*-values from one sample t-test (H_0_: μ = 0 and H_1_: μ ≠ 0) on protein fold changes relative to N2; *P ≤ 0.05; **P ≤ 0.01; ***P ≤ 0.001; ****P ≤ 0.0001. (B) Tukey-style box plot of broad-sense heritability for the 44 proteins shown in panel A. Scatter points overlaid on the box plot represent the broad-sense heritability values for the individual proteins (solid black circles correspond to selected proteins). (C) Bar graph of selected proteins from panel A. Horizontal dashed lines represent the fold change cut-off of 1.3 (~ 0.38 on log_2_ scale). Error bars represent SEM between three biological replicates.

**Fig 4:**
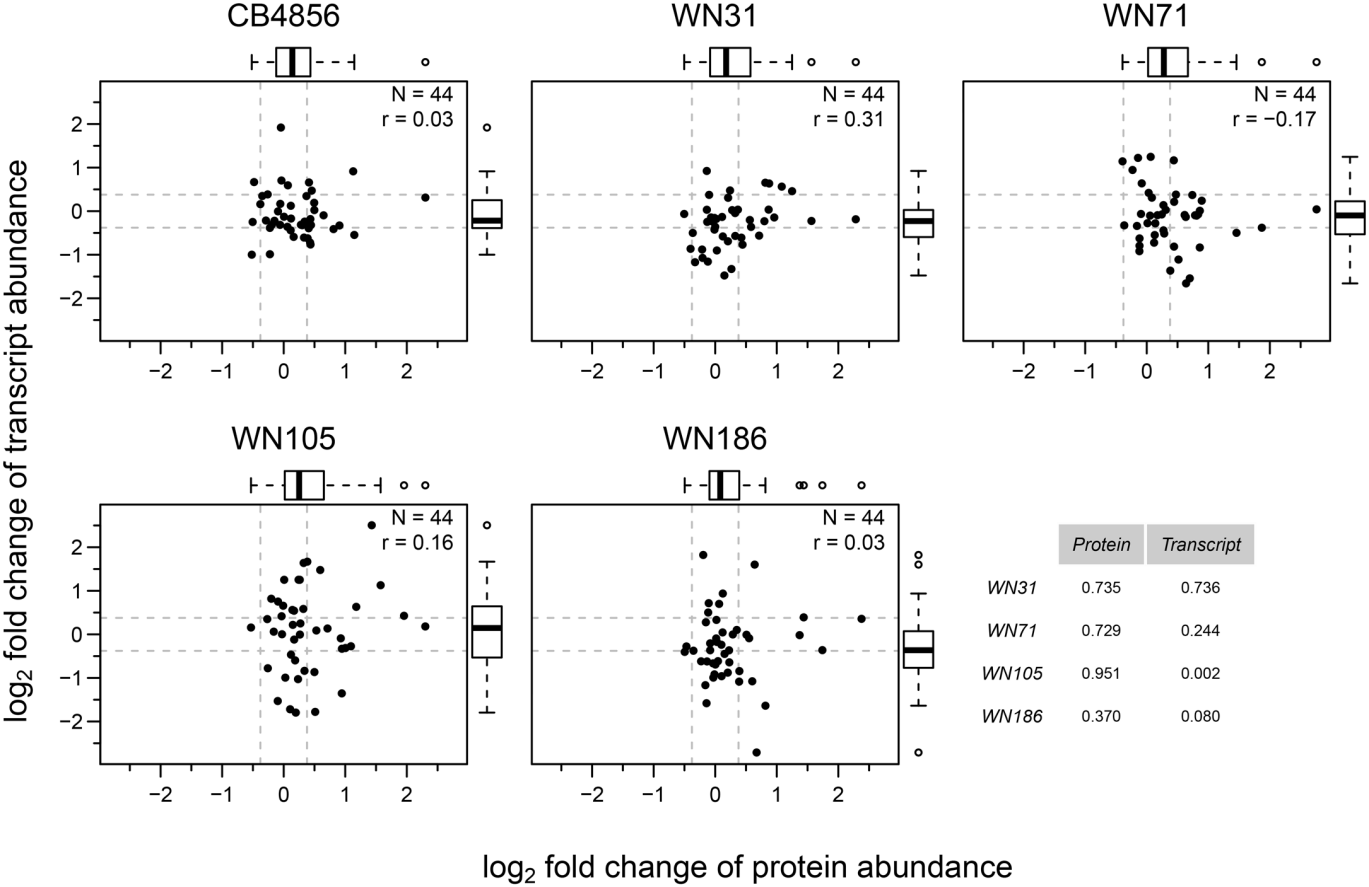
RILs show similar protein and transcript abundance variation for the tested 44 genes as the parental strains. Comparison of protein and transcript abundance (log_2_ scaled fold changes relative to N2) for 44 signalling pathway proteins in CB4856 and four selected RILs. Horizontal and vertical dashed lines represent the fold change cut-off of 1.3 (~ 0.38 on log_2_ scale). Tukey-style box plot on top and right side represents variability in protein and transcript log_2_ fold changes respectively. Pearson correlation coefficient is denoted by r. Table on bottom right represents the *P*-values from the Fligner-Killeen test for homogeneity of variances between protein (column 1) and transcript (column 2) data for RILs compared with CB4856.

Between 71 and 85 proteins (out of 148 proteins measured) were quantified by SRM in the four selected RILs; 44 proteins (represented by 114 peptides) were quantified in all four RILs. To gain more statistical confidence, we re-measured these 44 proteins (S2 Table) in the four selected RILs as well as in the two parental strains with three biological replicates (Fig 3A). Generally, the quantified proteins showed a trend to be up-regulated in CB4856 and in the RILs relative to N2 (*P* ≤ 0.01; Fig 3A), and about a quarter of the protein abundance changes were both statistically significant (*P* ≤ 0.05) and above the fold change cut-off of 1.3 (~ 0.38 on log_2_ scale; Fig 3A and S6 Fig). To determine how much of the observed variation in protein levels could be attributed to genetic variation, we calculated the broad-sense heritability [39] for the 44 proteins and found that on average, about half of the measured protein level variation was due to genetic variation (Fig 3B and S1 Table). We tested the extent of variation in abundance of the 44 protein, at both the transcript and protein level (relative to N2) using the Fligner-Killeen test for homogeneity of variances. No significant variation could be detected except between CB4856 and WN105 at the transcript level (Fig 4). Indicating that for this smaller set of 44 genes, the tested RILs do not show greater overall levels of expression, at the transcript and protein levels compared to the parental strains.

### Protein QTL mapping

**Fig 5:**
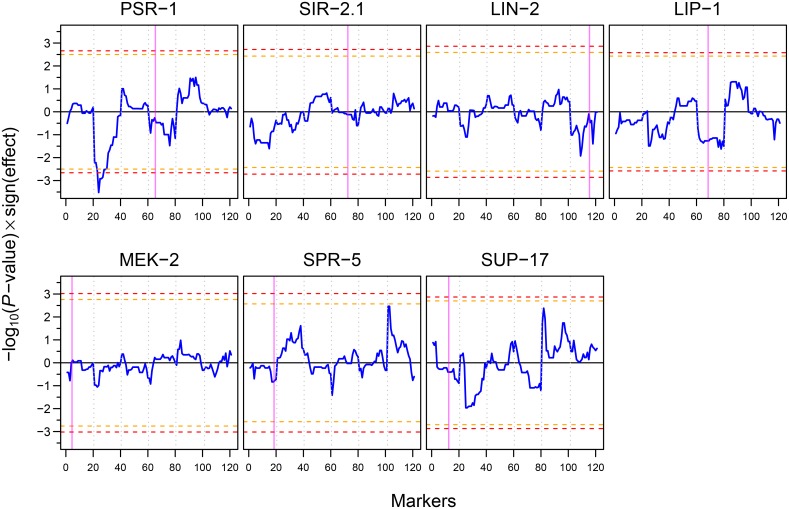
pQTL profiles of seven selected signalling pathway proteins. Blue curves show the significance of the pQTLs multiplied by the sign of the effect of the N2 allele (positive values of blue curve indicate higher protein abundance when the N2 allele is present, whereas negative values indicate higher protein abundance when the CB4856 allele is present). Horizontal orange and red dashed lines show 0.1 and 0.05 FDR thresholds respectively. Vertical dotted grey lines separate chromosomes I to V and X from left to right. Vertical magenta bands indicate the position of the gene in the genome. PSR-1 shows a significant pQTL on the left arm of chromosome II.

Most of the 156 proteins that we selected for SRM analysis have weak eQTLs at the transcript level (S1 Table) [27,40,41]. To explore the genetic basis of the protein abundance differences between parental strains and RILs, and to determine potential regulatory genes associated with the observed protein abundance differences, we performed protein quantitative trait locus (pQTL) mapping on seven proteins (PSR-1, SIR-2.1, LIN-2, LIP-1, MEK-2, SPR-5, and SUP-17; represented by 14 peptides; Figs 3A, 3C and S6) that showed significant abundance differences and fold changes above 1.3 among the six strains (two parental strains and four selected RILs). This mapping was used to determine whether genetic variation in the gene itself (or nearby location) was responsible for the observed protein abundance changes (local pQTL) or a change in another region away from the gene (distant pQTL) [42]. Mapping was done by linking the variation in abundances of the seven selected proteins (S2 Table) within the parental strains and 48 RILs (the four selected RILs and 44 additional ones; S7 Fig) with the genotypic variation in the same samples. For one of the seven proteins, PSR-1 (phosphatidylserine receptor), we found a significant distant pQTL on the left arm of chromosome II: the presence of the CB4856 allele at this position strongly correlated with a high PSR-1 protein abundance (Fig 5). Interestingly, this distant QTL is not apparent at the transcript level [24,29,43], suggesting that it likely affects a late step in the gene expression process (e.g. translation efficiency or protein stability). Early work in mouse [44] and yeast [45] showed that most of the pQTLs did not have corresponding variation at the transcript level, in contrast to this a much better agreement was found between eQTLs and corresponding pQTLs [46]. Recent studies in yeast using a new experimental design to overcome the sample size limitation of previous studies also suggest that over half of the previously reported eQTLs have corresponding pQTLs [47,48].

**Fig 6:**
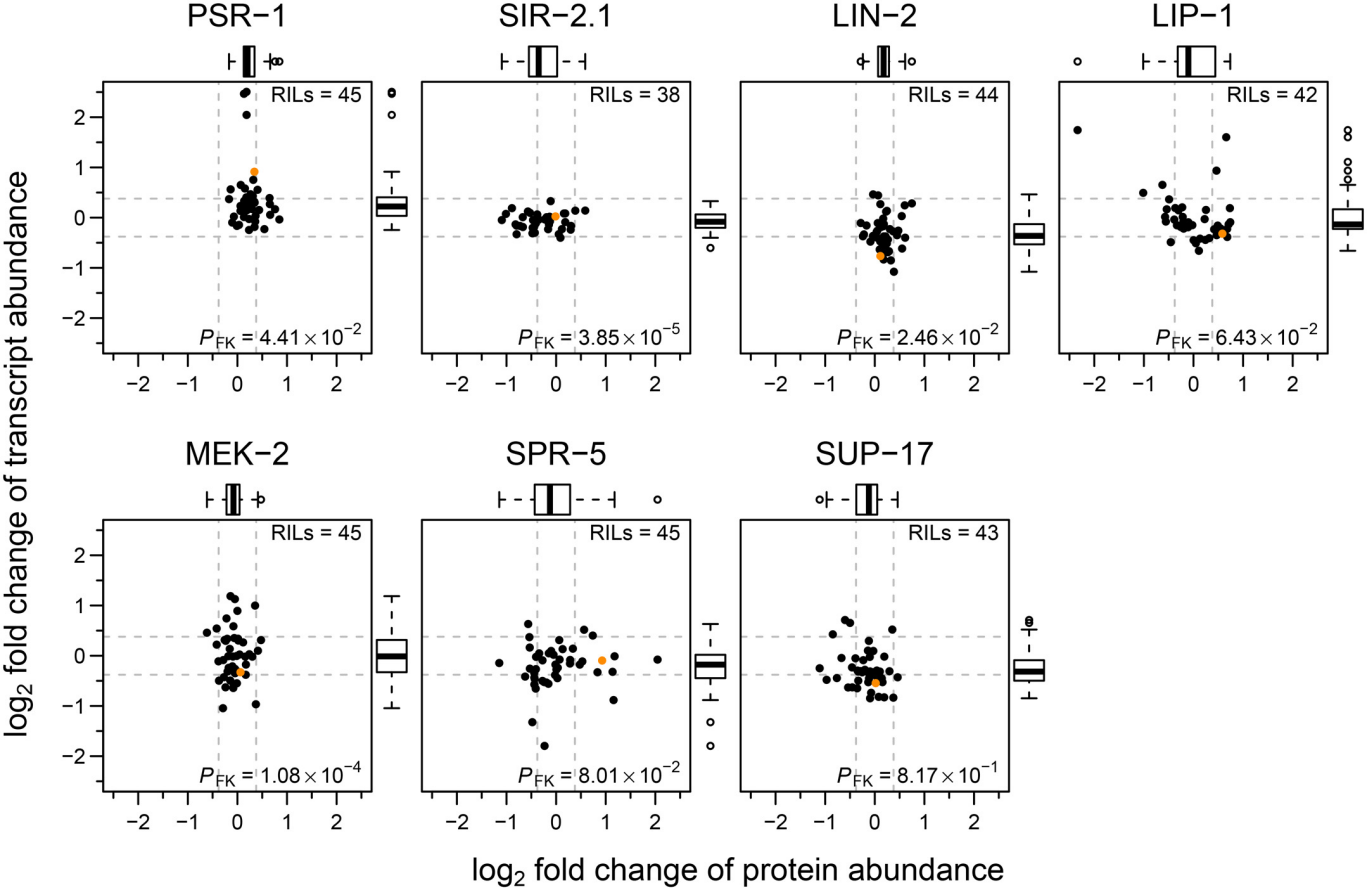
Protein and transcript abundance variation of the signalling pathway proteins used in pQTL mapping in CB4856 and 45 RILs. Comparison of protein and transcript abundance (log_2_ scaled fold changes relative to N2) in CB4856 (solid orange circle) and RILs for the seven proteins used in pQTL mapping. Horizontal and vertical dashed lines represent the fold change cut-off of 1.3 (~ 0.38 on log_2_ scale). Tukey-style box plot on top and right side represents variability in protein and transcript log_2_ fold changes respectively. *P*-value from Fligner-Killeen test for homogeneity of variances between protein and transcript data is denoted by *P*_FK_.

The large number of RIL measurements also allowed us to compare in more detail the relative variations in transcript and protein abundances for the seven selected proteins (Fig 6). Whereas some proteins (PSR-1, LIN-2, and MEK-2) showed a tendency for greater variation at the transcript level, SIR-2.1 showed a greater protein abundance variation while the remaining genes showed similar variation, at both protein and transcript levels.

### Natural variation in apoptosis levels

**Fig 7:**
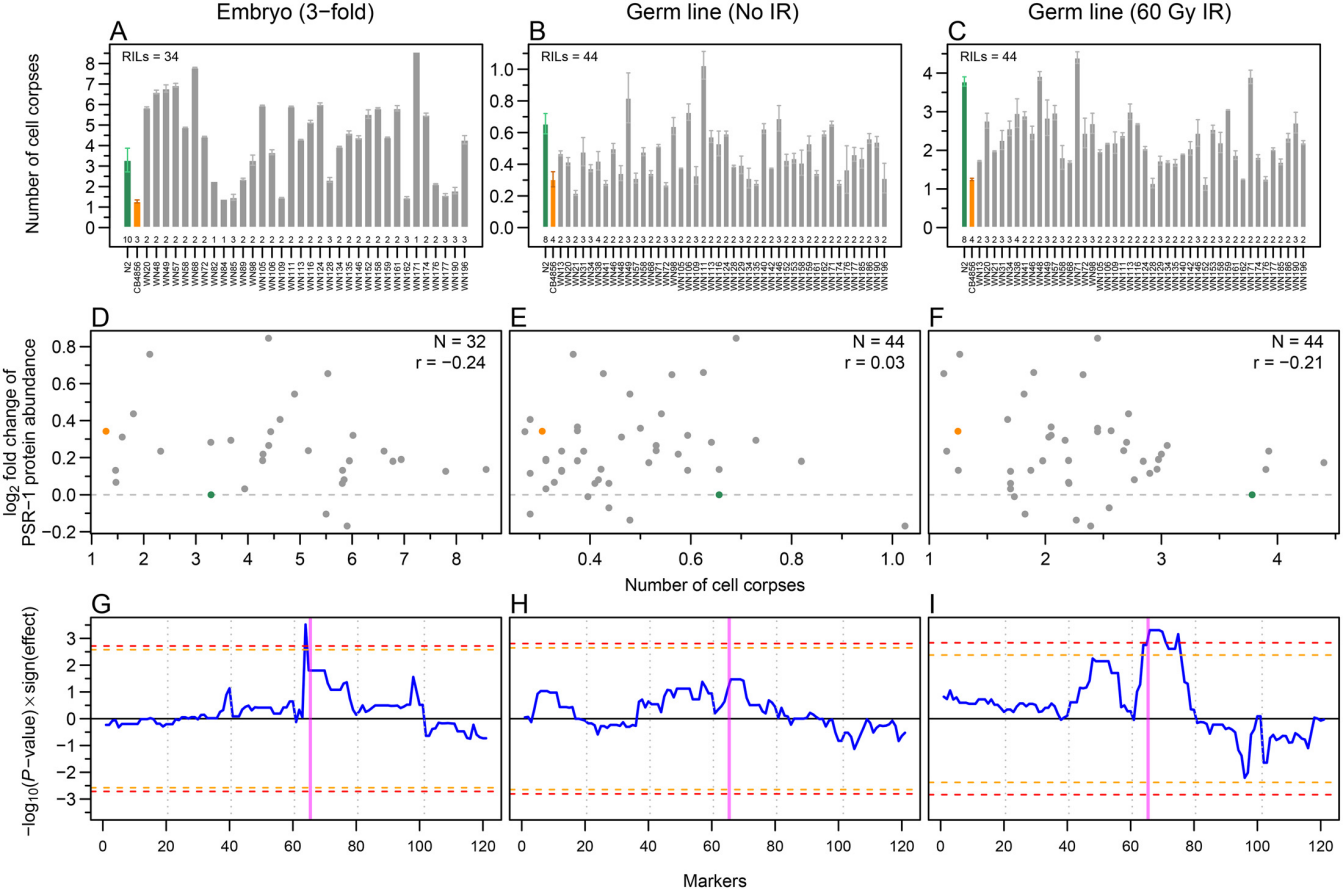
Analysis of variation in embryonic and germ line apoptosis in parental strains and RILs. (A-C) Quantification of apoptotic cell corpse numbers in embryos (A), germ line without ionizing radiation (IR; B), and with 60 Gy IR (C). Error bars represent SEM between numbers of biological replicates indicated at the bottom of each bar. (D-F) Natural variation in apoptosis levels of parental strains and RILs does not correlate with the PSR-1 protein abundance (relative to N2). Scatter plots (CB4856 in orange and N2 in green) of PSR-1 protein abundance and apoptotic levels in embryos (D), germ line without IR (E), and with 60 Gy IR (F). Pearson correlation coefficient is denoted by r. (G-I) Embryonic and IR-induced apoptosis shows a significant QTL on chromosome IV. QTL profiles of apoptotic phenotype in embryos (G), adult germ line without IR (H) and with 60 Gy IR (I). Blue curves show the significance of the QTLs multiplied by the sign of the effect of the N2 allele (positive values of blue curve indicate higher apoptosis level when the N2 allele is present, whereas negative values indicate higher apoptosis level when the CB4856 allele is present). Horizontal orange and red dashed lines show 0.1 and 0.05 FDR thresholds respectively. Vertical dotted grey lines separate chromosomes I to V and X from left to right. Vertical magenta band indicates the position of *psr-1* gene in the genome.

The PSR-1 protein has been implicated in the clearance of apoptotic cells in *C*. *elegans* [49,50]. To determine whether the changes in PSR-1 protein abundance might affect corpse clearance efficiency, we quantified both developmental [51] and germ line (physiological and DNA-damaged induced) [52] apoptosis levels, by counting apoptotic cell corpse numbers, both in embryos and in the adult germ line (Fig 7A–7C). Cell corpse numbers were consistently lower in CB4856 compared to N2. Although most RILs showed apoptotic cell corpse numbers between the two parental strains, some showed more extreme apoptotic levels than the parental strains (higher than N2, or lower than CB4856).

Unfortunately, we did not find any correlation between changes in PSR-1 protein abundance and apoptosis levels among the strains tested (Fig 7D–7F). Given that even the complete loss of PSR-1 function only results in a very mild change in cell corpse clearance [49], this result is not unexpected. We conclude that the variation in apoptosis observed between the RILs must arise from other genetic changes present between N2 and CB4856.

QTL mapping of the apoptosis levels revealed the presence of a QTL that affects ionizing radiation (IR)-induced apoptosis on chromosome IV and a QTL on the left arm of chromosome IV that affects embryonic cell death (in both cases, the presence of the N2 allele results in increased cell corpse numbers), whereas no QTLs were detected for physiological germ cell apoptosis (Fig 7G–7I). The QTL for IR-induced germ cell death is very broad, and might conceivably even consist of two (or more) linked QTLs on chromosome IV. By contrast, the embryonic cell corpse QTL is very sharp and maps close to, but can clearly be separated from, the physical position of *psr-1*. Interestingly, this region includes several genes that have been linked to apoptosis or cell corpse cleaning, including the engulfment gene *ced-10* [53] and the E3 ubiquitin ligase *eel-1* [54]. There are 8 and 20 SNPs between CB4856 and N2 for these two genes. While none of the *ced-10* SNPs change the protein sequence, one of the 20 SNPs in *eel-1* induces an amino acid change. Further analysis will be required to determine if any of those SNPs are responsible for the observed QTL in embryonic cell corpse apoptosis.

## Conclusion

Here we developed and made available a SRM method for 148 *C*. *elegans* signalling pathway proteins represented by 295 peptides.

Our quantitative analysis of protein abundance changes across six strains revealed that statistically significant changes can be found at a fair frequency (in about half of the cases), but that most of these changes are very minor in magnitude (less than 1.3-fold). This is not surprising, as we assume that many of the tested proteins should be under significant evolutionary pressure, which likely precludes large changes in protein abundance. Nevertheless, we also identified a smaller number of genes that showed reproducible protein abundance changes in the two- to fourfold. QTL analysis on a selected set of seven proteins that showed particularly strong changes allowed us to identify a potential distant pQTL on the left arm of chromosome II for PSR-1, a protein that intrinsically acts as receptor for phosphatidylserine exposed by apoptotic cells [49,50]. We also found two separate QTLs on chromosome IV affecting embryonic and IR-induced apoptosis levels, but failed to detect any correlation between PSR-1 abundance and apoptosis levels suggesting the involvement of other genes in these events.

## Materials and Methods

### Strains

We used *C*. *elegans* wild-type strains N2 [20] and CB4856 [23], and a set of 54 RILs (S3 Table) generated from them [24–27]. Unless otherwise stated all experiments were performed on synchronized developmental stage L4 worms, grown as previously described [7] on nematode growth medium (NGM) agar plates seeded with *Escherichia coli* strain OP50 at 20°C.

### Transcriptomics

From one sample of N2, CB4856 and 47 RILs (S3 Table) RNA extraction was done using RNEasy Micro Kit (Qiagen), as previously described (see “*RNA isolation*” from “Supplementary Materials and Methods” of [31]). Two-colour microarray was used to analyse the extracted RNA using 413 different hybridization probes (representing the 148 proteins of interest), as previously described (see “*Microarray sample preparation*, *scanning and normalization”* from “Supplementary Materials and Methods” of [31]) with the exception that we averaged normalised intensities from single channel for all probes of a gene, followed by the calculation of the log_2_ scale deviation of the gene expression value from the mean across all samples.

### Protein Extraction

Protein extraction was done as previously described [55] with minor changes. Briefly worm pellets were homogenized with glass-beads (G1277; Sigma-Aldrich) in lysis buffer (8 M urea, 50 mM Tris-HCl pH 8.3) in a 1:1:2 ratio using a benchtop homogenizer (FastPrep-24; MP Biomedicals) at 4°C, four times for 30 s at the speed of 5 m/s. After homogenization 0.125% SDS (v/v of buffer) was added and the homogenate was incubated at room temperature for 1 h to extract proteins. Proteins were separated from cell debris by centrifugation, and the protein concentration was determined using the Bradford reagent (B6916; Sigma-Aldrich).

### Database used for peptide spectrum matching

The *C*. *elegans* protein database wormpep212 (downloaded on March 2010, 24362 entries) was used for peptide spectrum matching. A decoy database generated by reversing the sequences was appended to forward database prior to the search, to facilitate the calculation of false discovery rate (FDR) [56], followed by addition of 259 common mass spectrometry contaminants yielding a total of 48983 entries.

### Sample Preparation for SRM

#### Selection and solubilisation of hPTPs

For each protein of interest, 1–5 tryptic proteotypic peptides (PTPs [57]; without cysteine and methionine; in total 377 PTPs; S2 Fig and S1 Table) were selected based on a *C*. *elegans* shotgun dataset [55]. These 377 PTPs were synthesized with isotopically labelled (^13^C and ^15^N) C-terminal Arginine and Lysine (SpikeTide L; JPT Peptide Technologies GmbH) as heavy labelled PTPs (hPTPs) for internal controls for SRM method development and SRM experiments. Prior to spike-in, hPTPs were dissolved in 180 μl of 20% ACN and 1% FA solvent. Either 0.4 nmol or 4 nmol of each peptide (depending on whether the abundance of the corresponding protein from PaxDb version 2.1 [34] is less than 100 ppm or greater than 100 ppm) was mixed to generate a 100× hPTPs master-mix. Separate 100× hPTPs master-mixes were prepared for experiments targeting 148, 44, and 7 proteins with including only peptides corresponding to those proteins.

#### Protein digestion and peptide pre-fractionation

250 μg of total worm protein from each sample was mixed with a 100 times diluted hPTP master-mix and digested overnight with trypsin (V5111; Promega) in the ratio of 50:1 (protein:trypsin) in digestion buffer (8 M urea, 50 mM Tris-HCl pH 8.3, 0.125% SDS, pH 7–8) at 37°C. Anionic SDS was removed after digestion using strong cation exchange (SCX). Samples were applied on SCX cartridges (Applied Biosystems) in loading buffer (10 mM KH_2_PO_4_, 25% ACN, pH < 3.0), and after washing peptides were eluted with 1 ml of elution buffer (10 mM KH_2_PO_4_, 25% ACN, 350 mM KCl, pH < 3.0). The cartridge was cleaned between subsequent samples using cleaning buffer (10 mM KH_2_PO_4_, 25% ACN, 1 M KCl, pH < 3). Salt was removed by solid phase extraction (SPE). Samples were applied on C18 SPE columns (Finisterre^™^, Teknokroma) in loading solution (5% ACN, 0.1% TFA), and after washing peptides were eluted with 1 ml of elution solution (60% ACN, 0.1% TFA). To circumvent the problem of inefficient ionization of target peptides in the complex background of the total worm protein extract, we used offline pre-fractionation by reversed phase-high pressure liquid chromatography (RP-HPLC) at pH 11.0 on an Agilent 1100 series HPLC system using a YMC Triart C18 column (150 mm × 4.6 mm ID, particle size 5 μm, pore size 12 nm) at a flow rate of 1 ml/min. Peptide samples were applied in buffer A (20 mM K_2_HPO_4_, 5% ACN, pH 11.0), and were eluted with a gradient between buffer A and buffer B (20 mM K_2_HPO_4_, 50% ACN, pH 11.0) into 47 fractions (pooled to 10 final fractions). The gradient profile was 2% buffer B between 0–20 min, 2%-50% buffer B between 20–50 min, 50%-98% buffer B between 50–55 min and 98% buffer B between 55–60 min followed by column re-equilibration to 2% buffer B in a total 80 min run. Buffer A and Buffer B were prepared from the stock solution (200 mM K_2_HPO_4_, pH 11.0).

For the pQTL experiments, peptide samples (directly after tryptic digestion) were pre-fractionated by hydrophilic interaction liquid chromatography (HILIC) on an Agilent 1100 series HPLC system using a YMC-pack polyamine II column (250 mm × 4 mm ID, particle size 5 μm, pore size 12 nm) at a flow rate of 0.85 ml/min. Peptide samples were applied in buffer A (8 mM KH_2_PO_4_, 75% ACN, pH 4.5), and were eluted with a gradient between buffer A and buffer B (100 mM KH_2_PO_4_, 5% ACN, pH 4.5) into 14 fractions (pooled to 3 final fractions). The gradient profile was 0% buffer B between 0–7.5 min, 0%-50% buffer B between 7.5–37.5 min, 50%-100% buffer B between 37.5–42.5 min and 100% buffer B between 42.5–47.5 min followed by column re-equilibration to 0% buffer B in a total 67.5 min run. Buffer A and buffer B were prepared from the stock solution (400 mM KH_2_PO_4_, pH 4.5).

To identify in which fractions the target peptides were eluted, all fractions were analysed by shotgun proteomics.

### SRM method development and analysis

#### Generation of spectral library

For each peptide two transitions were selected for doubly and triply charged precursor ions using Skyline [58] with the following transition settings (Precursor charges = 2 and 3, Ion charges = 1, Ion types = y, Product ions from = m/z > precursor, Product ions to = 2, and Always add = N-Terminal to Proline enabled). Transitions with |Q3-Q1| m/z < 40 were removed to reduce interferences. Remaining transitions were used to acquire MS/MS spectra triggered by SRM (SRM-triggered MS/MS [32,59]) on a TSQ Vantage (Thermo Scientific) triple quadrupole mass spectrometer (MS) coupled with a nanoLC-ultra chromatography instrument (Eksigent Technologies). Peptides were separated on a self-packed C-18 (Magic C18 AQ, particle size 3 μm, pore size 20 nm; Michrom) column (15 cm × 75 μm ID) at a flow rate of 200 nl/min. Peptides were loaded in 3% ACN, 0.1% FA and were eluted with a gradient between solvent A (water with 0.1% FA; Biosolves) and solvent B (ACN with 0.1% FA; Biosolves). The gradient profile was 5%-40% buffer B between 0–56 min, 40%-47% buffer B between 56–60 min, 47%-97% buffer B between 60–64 min and 97% buffer B between 64–71 min followed by column re-equilibration to 5% buffer B in a total 80 min run. The MS was operated in SRM mode (Q1_FWHM_ = 0.40, Q3_FWHM_ = 0.70, and dwell time = 20 ms with roughly 240 transitions per run), triggering MS/MS acquisition (Q1_FWHM_ = 0.70, Q3_FWHM_ = 0.70, and dwell time = 50 ms) if it detected counts higher than 300. MS/MS spectra were searched against the *C*. *elegans* protein database wormpep212 using the Mascot search engine (version 2.3.02; Matrix Science) [60], with the following search parameters (Type of search = MS/MS ion search, Enzyme = Trypsin with zero missed cleavage, Variable modifications = Arg10 and Lys8, Peptide mass-tolerance = 2 Da, and Fragment mass-tolerance = 0.8 Da). This resulted in the identification of 356 (94.4%) peptides with a 3.3% FDR at peptide level.

The Mascot search results in mascot DAT file format were used to build a MS/MS spectra library for 340 (90.2%) peptides corresponding to 154 proteins using Skyline with a score cut-off setting of 0.95. From this MS/MS spectra library, either doubly or triply charged precursor ions were selected for each peptide and for each selected precursor the five most intense y-ions fulfilling the following criteria (precursor m/z > 400 and |Q3-Q1| m/z > 5) were selected to generate the final transition list 2950 transitions, including both heavy and light isoforms (S2 Fig and S2 Table).

#### SRM

After peptide pre-fractionation, ten fractions from RP-HPLC at pH 11.0 or three fractions from HILIC per strain were desalted by using ZipTip C18 (Millipore). Samples were applied at a speed of 1 drop/s in washing solution (5% ACN, 0.1% TFA; sample pH was adjusted below 3, if necessary with 5% ACN, 10% TFA), and after washing peptides were eluted with 80 μl of elution solution (60% ACN, 0.1% TFA) at a speed of 1 drop/s. Peptide samples were completely dried in a centrifugal evaporator and dissolved in 3% ACN, 0.1% FA solvent. SRM measurements were also performed on a TSQ Vantage (Thermo Scientific) coupled with a nanoLC-ultra (Eksigent Technologies). The setup was as described above except that the MS was operated in scheduled SRM (sSRM) mode (Q1_FWHM_ = 0.40, Q3_FWHM_ = 0.70, and cycle time = 2 s with a retention time window of 10 min for each peptide). For each off-line HPLC fraction a separate method containing only transitions for target peptides in that fraction was used. iRT kit (Biognosys) peptides were used as retention time standard [61].

### SRM data analysis

We used the mProphet software (version 1.0.4.1) [62] for the identification of true peak groups among each transition group record from SRM data, followed by a protein significance analysis using the intensity-based linear mixed-effects model [63] implemented in R [64] based on the package MSstats (version 0.99.0 for Fig 3A and version 1.99.0 for pQTL experiments) [65].

Briefly, at first raw data from the MS were converted into the mzXML file format [66] using command line tool ReAdW (version 4.0.2, ISB/SPC) with default options. Within mProphet, these mzXML files were mapped to transition lists using the mMap module (with parameter file = param_AQUA_heavy_stringent_ref_synthetic.def and machine type = QQQ) followed by peak picking using mQuest module (with parameter file = param_AQUA_heavy_stringent_ref_synthetic.def), and finally mQuest score optimization was done using the mProphet module (with workflow = SPIKE_IN). For each sample (based on mprophet_stat.xlsx file) an appropriate normalised discriminate score (d_score) cut-off was selected to keep the number of true positive peak groups higher and the number of false positive peak groups lower (for Fig 3A this was done by keeping a q-value of 0.05 and for pQTL analysis by keeping a q-value of 0.01). Finally, identified peak groups with a d_score lower than the selected cut-off and dummy peak groups were filtered out from the final output file of mProphet software (mProphet_peakgroups.xlsx).

For MSstats based analyses an input file (type “?RawData” in R console for details) with an intensity value for each transition of selected peak groups at both light and heavy isotopic level was generated using custom R scripts. To ensure that each peptide was analysed in the same fraction across all samples, we used fraction number concatenated with peptide as “PeptideSequence” in the input file. All fractions measured in one sample were considered to belong to the same “Run” under input file. Data analysis was done as previously described [63], considering interference in transitions and keeping “scope of biological variation” as restricted and “scope of technical variation” as expanded.

For S3 Fig, after mProphet analysis, Microsoft Excel and custom R scripts were used to sum intensity values for all transitions of a protein (including all detected peptides, if measured in all samples) at both light and heavy isotopic level separately for both replicates. These intensity values were appropriately scaled to make the median of resulted deconvoluted ratios (CB4856_Light_/CB4856_Heavy_)/(N2_Light_/N2_Heavy_) for proteins in both biological replicates equal to 1. The ratios of averages of scaled intensities of CB4856 and N2 from both replicates represent the average protein fold change in CB4856 relative to N2. Whereas log_10_ transformed intensities were used to perform a two-sample equal variance t-test, resulting *P*-values were corrected for multiple hypothesis testing using the Benjamini-Hochberg (BH) procedure [67] (type “?p.adjust” in R console for details).

### QTL analysis

The QTLs were mapped using a custom R script applying linear regression on a single marker model as previously described [68]. For pQTLs, we used the log_2_ scale abundance of seven selected proteins in one sample of CB4856 and 48 RILs (S3 Table) relative to N2 as phenotypic data and genotypic variation in same samples using 121 markers [24]. Thresholds were determined per trait by 1000 permutations, where the trait values for the individual samples were randomly distributed before QTL mapping. Comparison to previously published eQTL data was done by extracting the data from WormQTL (http://www.wormqtl.org) [27,40,41]. QTL mapping of apoptosis levels was done as described above where average corpse number in N2, CB4856 and RILs were considered as phenotypic data.

### Apoptotic cell corpse counting

For embryonic cell corpse counting, between 6 and 23 embryos (3-fold stage) from N2, CB4856 and 34 RILs (S3 Table; Fig 7A for number of biological replicates) were counted in the head region using differential interference contrast (DIC) microscopy, as previously described [69] but instead of worms, mixed staged embryos were used. For germ line cell corpse counting, between 15 and 25 synchronized young adult hermaphrodites (12 h post L4/adult molts) from N2, CB4856 and 44 RILs (S3 Table; Fig 7B and 7C for number of biological replicates) were either exposed to no ionizing radiation (No IR) or 60 Gy ionizing radiation (60 Gy IR). After 24 h germ line apoptotic cell corpses were counted by using DIC microscopy, as previously described [69].

### Heritability

Broad-sense heritability [39] was calculated in R using the following formula H^2^ = msq.geno/(msg.geno + msq.error), where msg.geno was the mean sum of squares of genotype (between variation) and msq.error was the mean sum of squares of error (within variation).

### Statistical analysis

Unless otherwise stated all statistical tests were performed in R using the package stats (version 3.2.2). Specifically, function “t.test” (type “?t.test” in R console for details) was used for one sample t-test and function “fligner.test” (type “?fligner.test” in R console for details) was used for Fligner-Killeen test for homogeneity of variances.

## Supporting Information

S1 Fig Signalling pathway proteins used in this study show a similar abundance distribution as the whole *C*. *elegans* proteome. Relative abundance data of *C*. *elegans* proteins were extracted from PaxDb version 2.1 [34]. (A) Data shown for 156 selected signalling pathway proteins. Each square represents a protein from one of four selected pathways, black squares (both solid and open) represent the 44 quantified proteins from Fig 3A; black open squares represent the 7 proteins (mostly under 20 ppm) selected for pQTL mapping. Parenthesis on top indicates number of proteins belonging to each pathway. (B) Histogram of all *C*. *elegans* proteins from PaxDb (top) and abundance distribution of the 156 selected signalling proteins (bottom, redrawn from A).
(TIF)

S2 Fig Outline of the SRM method development. See Materials and Methods for details.
(TIF)

S3 Fig The N2 and CB4856 parental strains do not show any strong protein abundance variation of the tested signalling pathway proteins. Protein abundance was quantified by SRM. Identification of the true peak group was performed using the mProphet software, followed by protein significance analysis using Microsoft Excel 2010 and custom R scripts. Horizontal dashed lines represent the fold change cut-off of 1.3 (~ 0.38 on log_2_ scale). Error bars represent SEM between two biological replicates. BH corrected *P*-values for all proteins from a two-sample equal variance t-test were above 0.9.
(TIF)

S4 Fig SILAC-based shotgun and SRM quantification show similar measurement accuracy. Scatterplots with Tukey-style box plot representing variation in measurement of protein abundance in CB4856 relative to N2, within two biological replicates using SRM (A) and between the averages of two biological replicates using SRM with three biological replicates using SILAC-based shotgun mass spectrometry data from [35] (B) Horizontal and vertical dashed lines represent the fold change cut-off of 1.3 (~ 0.38 on log_2_ scale). Pearson correlation coefficient is denoted by r. (C) Tukey-style box plot for protein abundance of 71 proteins using SRM, indicating that variation in protein abundance between CB4856 and N2 is not greater than between two biological replicates of one of the two parental strains. Vertical dashed lines represent the fold change cut-off of 1.3 (~ 0.38 on log_2_ scale). N2-1 is 1^st^ biological replicate of N2, N2-2 is 2^nd^ biological replicate of N2, CB4856-1 is 1^st^ biological replicate of CB4856, and CB4856-2 is 2^nd^ biological replicate of CB4856.
(TIF)

S5 Fig Correlation between difference in protein and transcript abundances between the parental strains. Scatterplots with Tukey-style box plot show log_2_ fold change for the tested signalling pathway proteins and transcripts in CB4856 relative to N2. Overall and pathway specific Pearson correlation coefficient is denoted by r.
(TIF)

S6 Fig Differential abundance of signalling pathway proteins in CB4856 and RILs relative to N2. Tukey-style box plot of protein abundance (log_2_ scaled fold changes relative to N2) redrawn from Fig 3A. Scatter points overlaid on the box plot represent the protein abundance values in CB856 and RILs. Most of the protein changes are either non-significant (P > 0.05) or below the fold change cut-off of 1.3 (~ 0.38 on log_2_ scale; horizontal dashed lines). Seven proteins (unfilled black boxes) with significant abundance differences and fold changes above 1.3 were selected for pQTL mapping.
(TIF)

S7 Fig Differential abundance of proteins selected for pQTL mapping in CB4856 and 48 RILs. Protein abundance was quantified by SRM. Identification of the true peak group was performed using the mProphet software, followed by protein significance analysis using an intensity-based linear mixed-effects model implemented in MSstats. Number of asterisks represent BH corrected *P*-values as follows, *P ≤ 0.05; **P ≤ 0.01; ***P ≤ 0.001; ****P ≤ 0.0001. Blue and red shades within heat map represent log_2_ scaled fold changes in protein abundance relative to N2 (white boxes = no data).
(TIF)

S1 Table Proteins and peptides used in this study. File with separate sheets for the list of 156 proteins (along with relative protein abundance data extracted from PaxDb version 2.1 and eQTL data extracted from WormQTL), broad-sense heritability values for 44 proteins, and the list of 377 PTPs.
(XLSX)

S2 Table SRM transitions used in this study. File with separate sheets for SRM transition lists (targeting 148, 44, and 7 proteins) used in this study and a sheet with normalised retention time (iRT) of peptides.
(XLSX)

S3 Table Strains used in this study. File with all the RILs along with parental strains N2 and CB4856 used in this study indicating (by “Yes”) the experiments performed on them.
(XLSX)

S4 Table List of abbreviations. (XLSX)
