# Supplementary figures and images for: Natural Genetic Variation Influences Protein Abundances in C. elegans Developmental Signalling Pathways
Source: PLoS One. 2016 Mar 17;11(3):e0149418. doi: 10.1371/journal.pone.0149418 (PMC4795773; doi:10.1371/journal.pone.0149418)

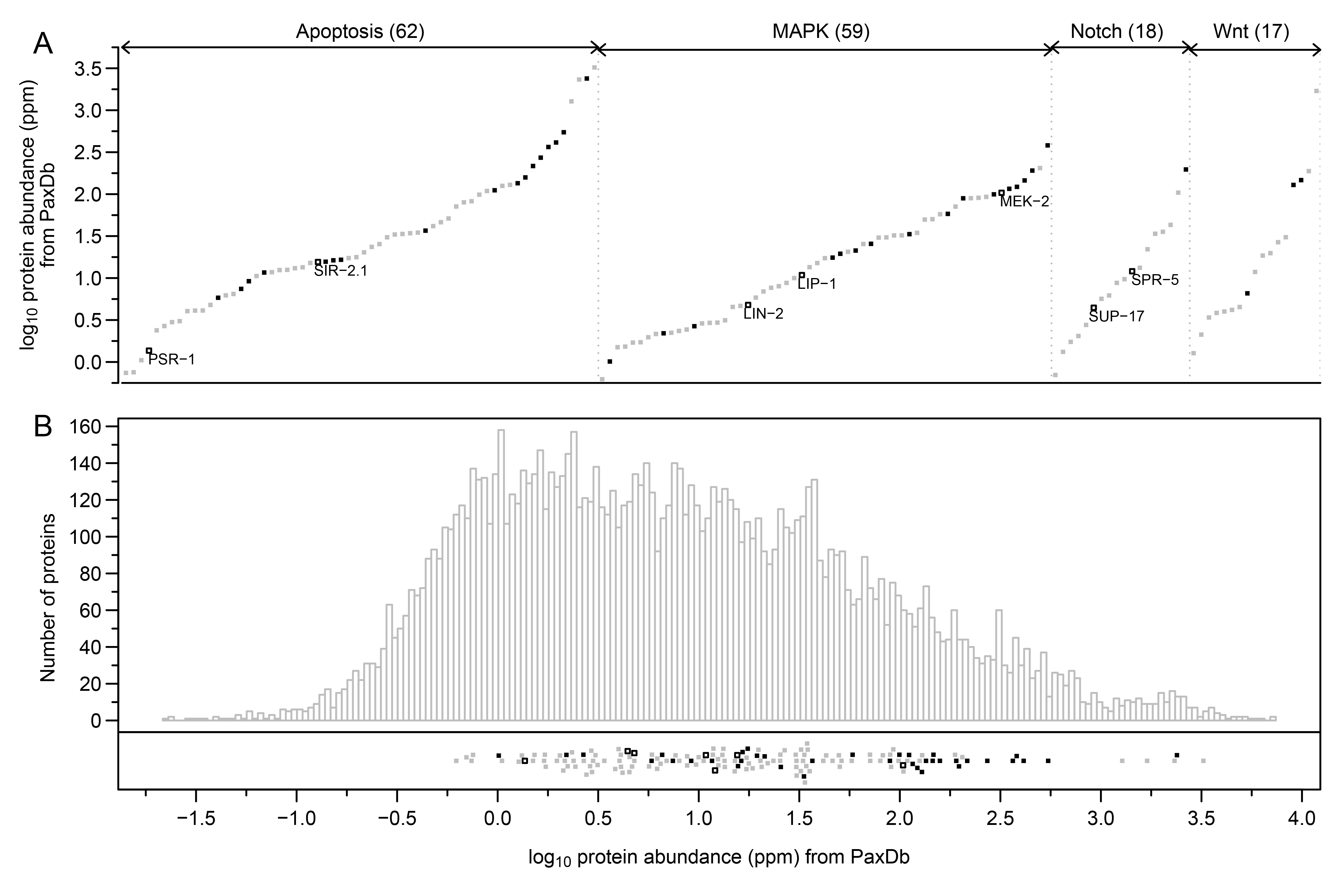

Supplement: S1 Fig — Relative abundance data of C. elegans proteins were extracted from PaxDb version 2.1 [34]. (A) Data shown for 156 selected signalling pathway proteins. Each square represents a protein from one of four selected pathways, black squares (both solid and open) represent the 44 quantified proteins from Fig 3A; black open squares represent the 7 proteins (mostly under 20 ppm) selected for pQTL mapping. Parenthesis on top indicates number of proteins belonging to each pathway. (B) Histogram of all C. elegans proteins from PaxDb (top) and abundance distribution of the 156 selected signalling proteins (bottom, redrawn from A). (TIF) [file pone.0149418.s001.tif]

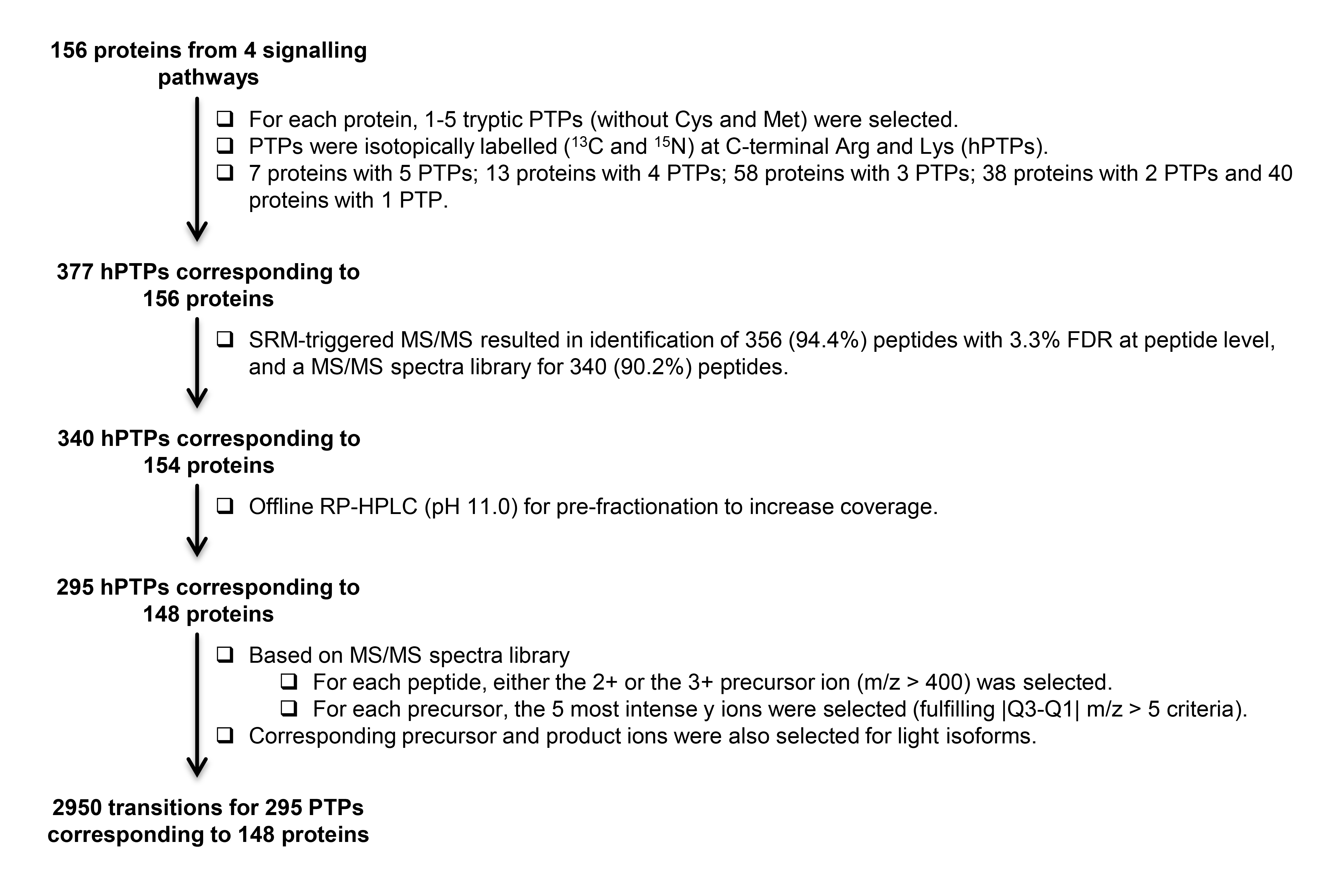

Supplement: S2 Fig — See Materials and Methods for details. (TIF) [file pone.0149418.s002.tif]

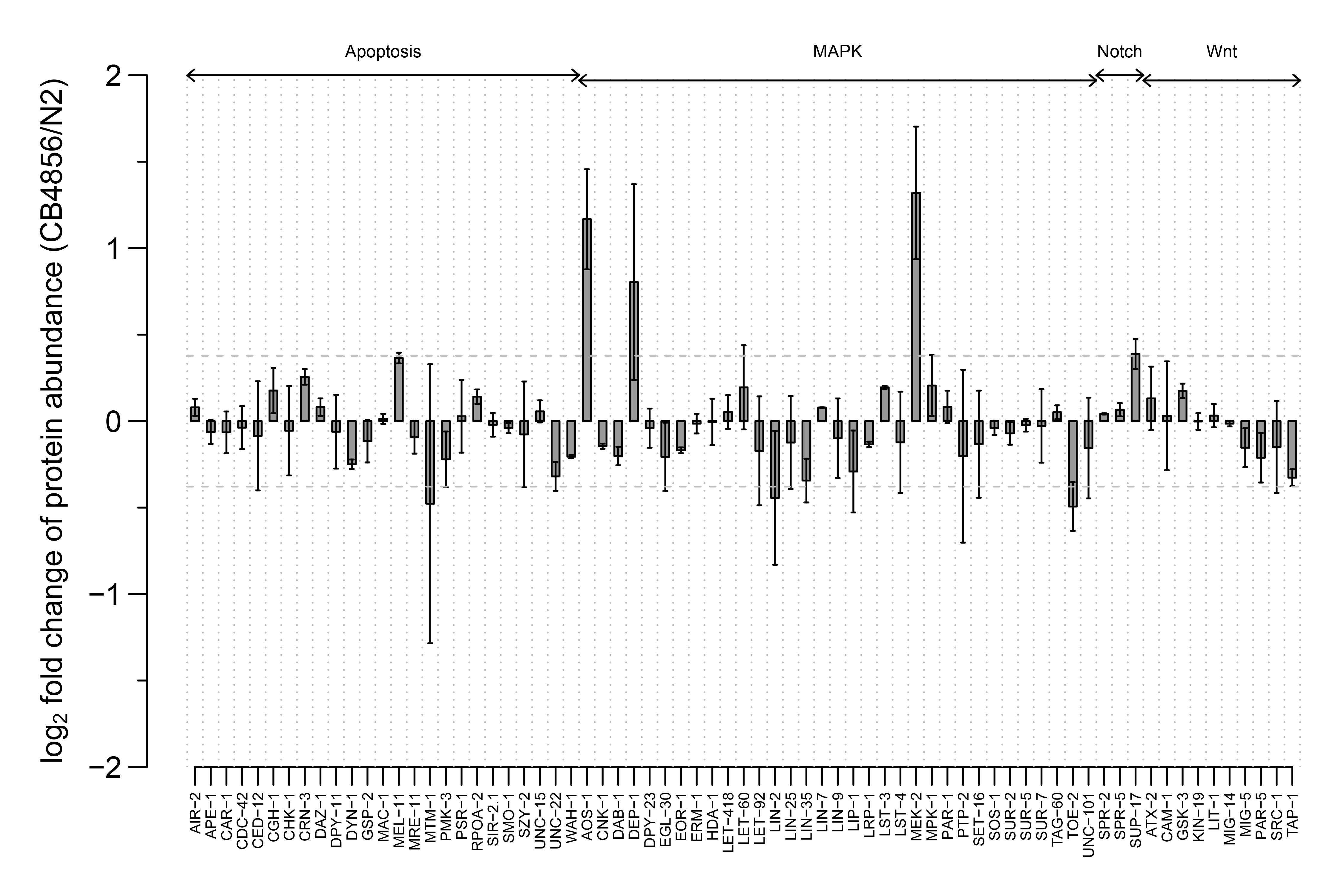

Supplement: S3 Fig — Protein abundance was quantified by SRM. Identification of the true peak group was performed using the mProphet software, followed by protein significance analysis using Microsoft Excel 2010 and custom R scripts. Horizontal dashed lines represent the fold change cut-off of 1.3 (~ 0.38 on log2 scale). Error bars represent SEM between two biological replicates. BH corrected P-values for all proteins from a two-sample equal variance t-test were above 0.9. (TIF) [file pone.0149418.s003.tif]

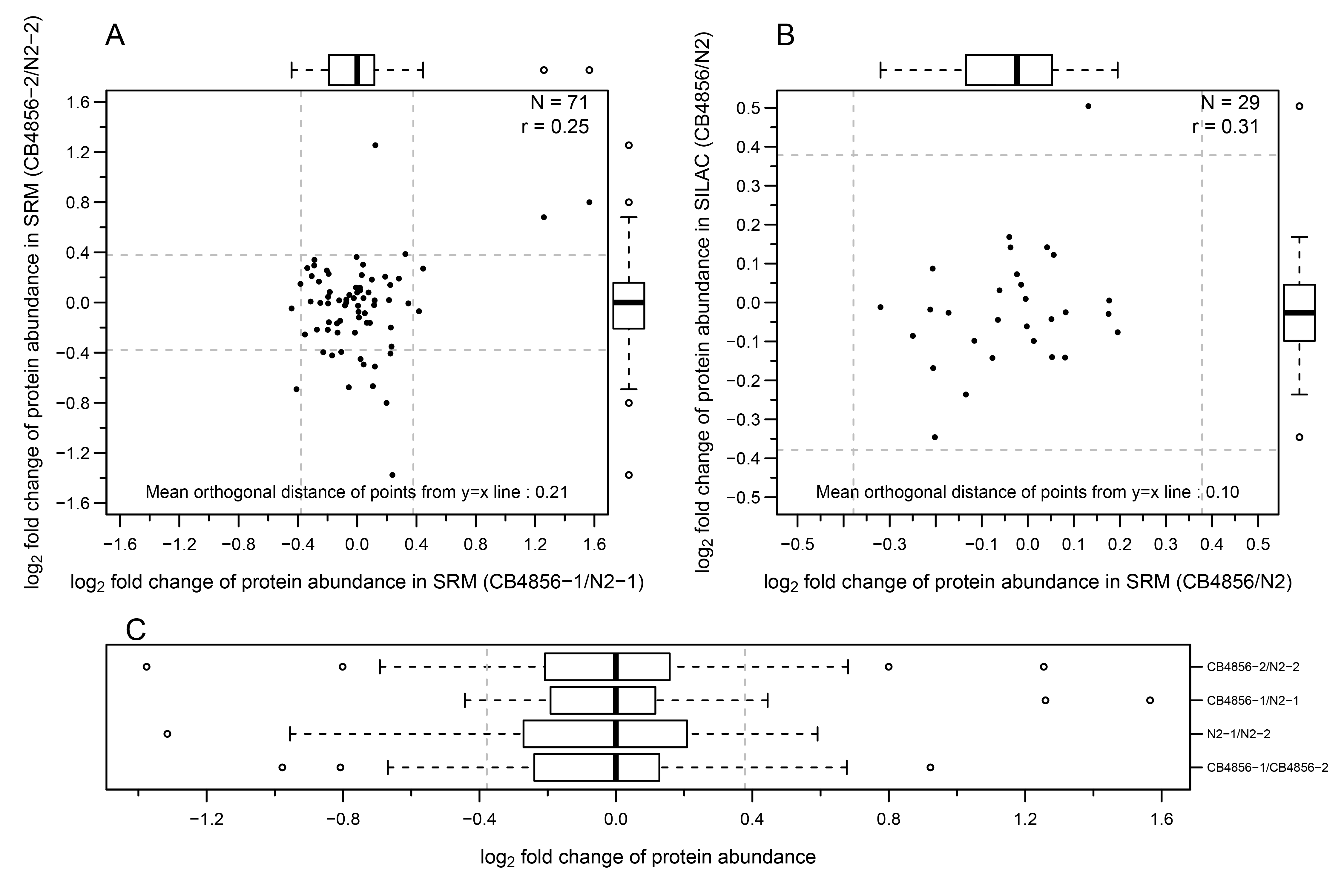

Supplement: S4 Fig — Scatterplots with Tukey-style box plot representing variation in measurement of protein abundance in CB4856 relative to N2, within two biological replicates using SRM (A) and between the averages of two biological replicates using SRM with three biological replicates using SILAC-based shotgun mass spectrometry data from [35] (B) Horizontal and vertical dashed lines represent the fold change cut-off of 1.3 (~ 0.38 on log2 scale). Pearson correlation coefficient is denoted by r. (C) Tukey-style box plot for protein abundance of 71 proteins using SRM, indicating that variation in protein abundance between CB4856 and N2 is not greater than between two biological replicates of one of the two parental strains. Vertical dashed lines represent the fold change cut-off of 1.3 (~ 0.38 on log2 scale). N2-1 is 1st biological replicate of N2, N2-2 is 2nd biological replicate of N2, CB4856-1 is 1st biological replicate of CB4856, and CB4856-2 is 2nd biological replicate of CB4856. (TIF) [file pone.0149418.s004.tif]

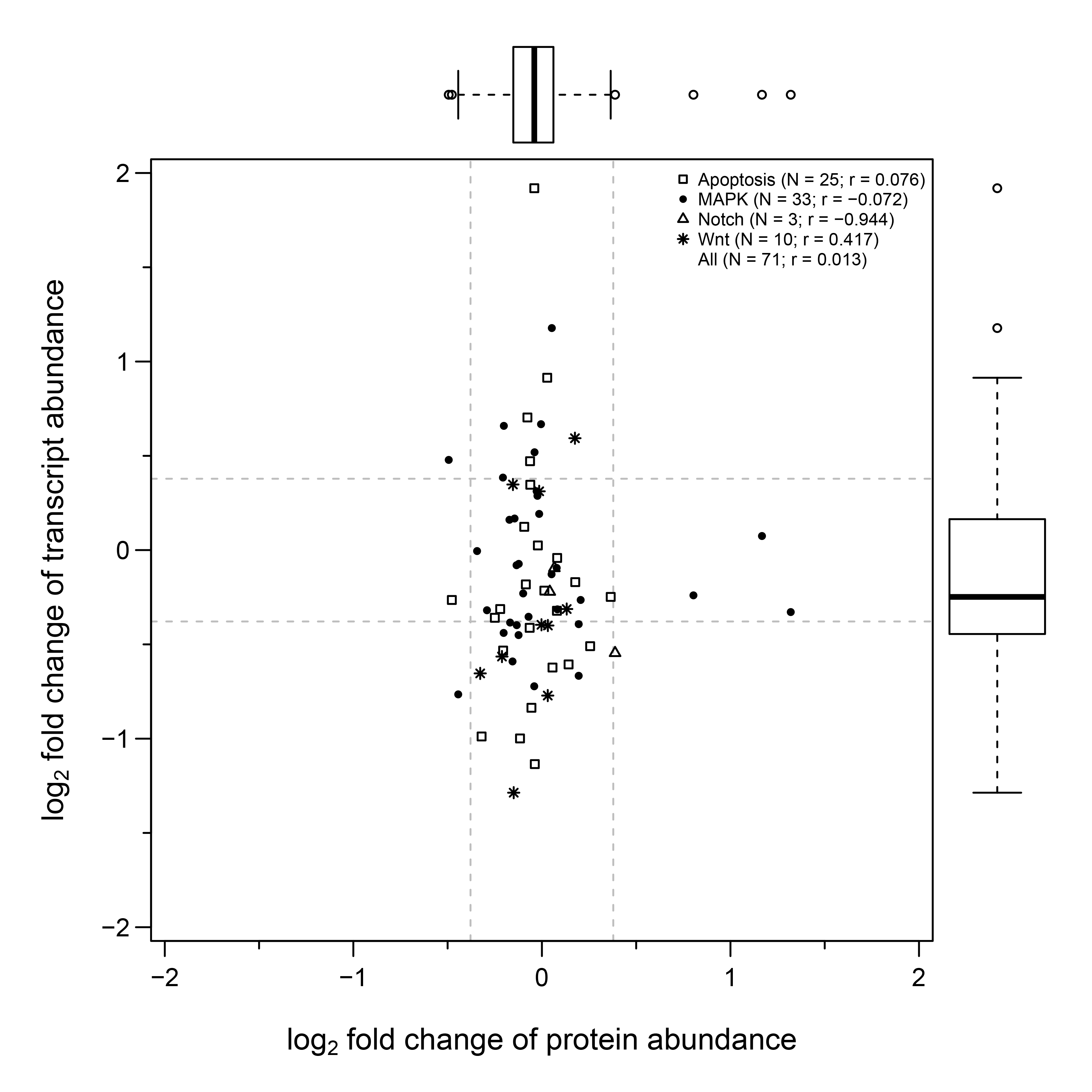

Supplement: S5 Fig — Scatterplots with Tukey-style box plot show log2 fold change for the tested signalling pathway proteins and transcripts in CB4856 relative to N2. Overall and pathway specific Pearson correlation coefficient is denoted by r. (TIF) [file pone.0149418.s005.tif]

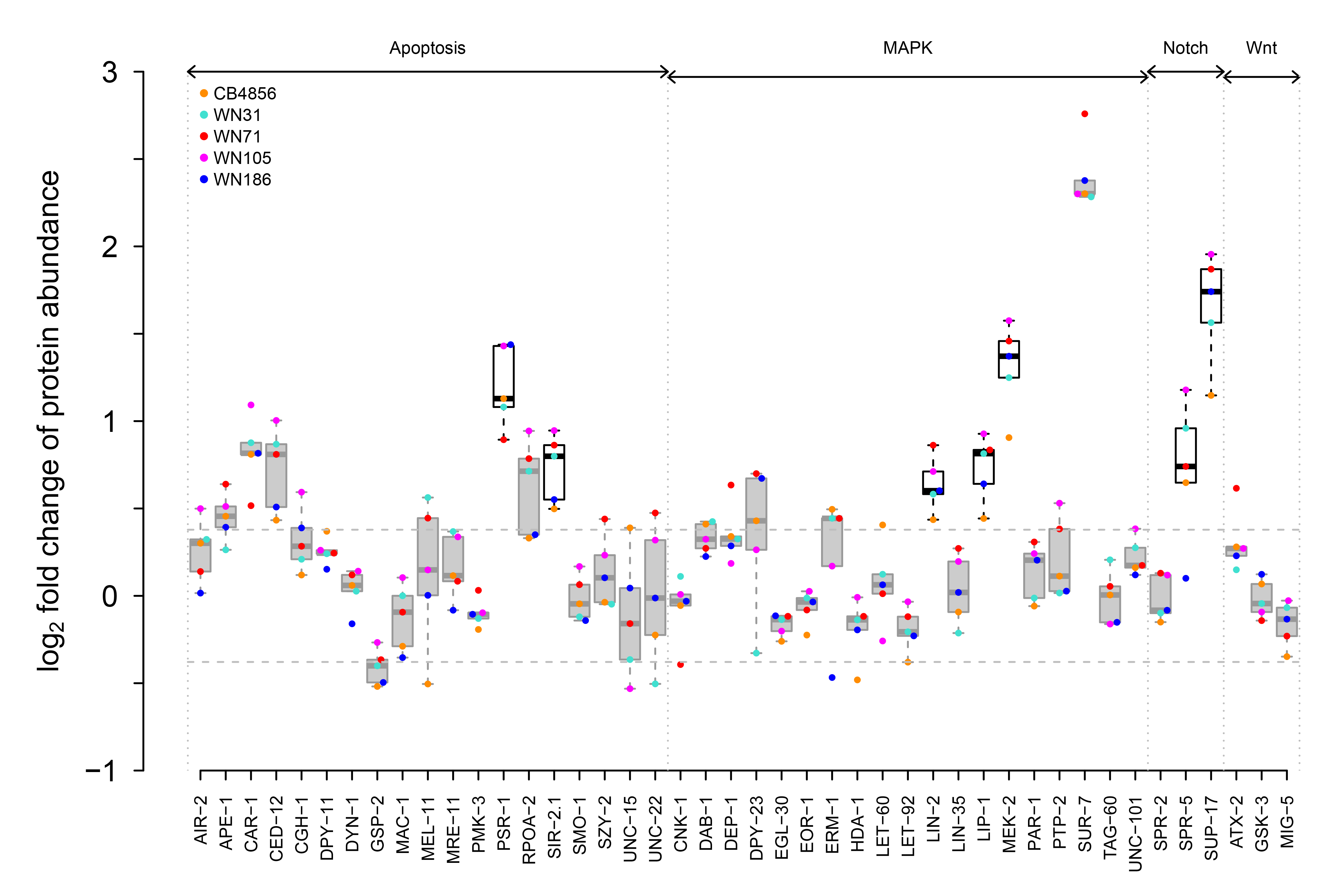

Supplement: S6 Fig — Tukey-style box plot of protein abundance (log2 scaled fold changes relative to N2) redrawn from Fig 3A. Scatter points overlaid on the box plot represent the protein abundance values in CB856 and RILs. Most of the protein changes are either non-significant (P > 0.05) or below the fold change cut-off of 1.3 (~ 0.38 on log2 scale; horizontal dashed lines). Seven proteins (unfilled black boxes) with significant abundance differences and fold changes above 1.3 were selected for pQTL mapping. (TIF) [file pone.0149418.s006.tif]

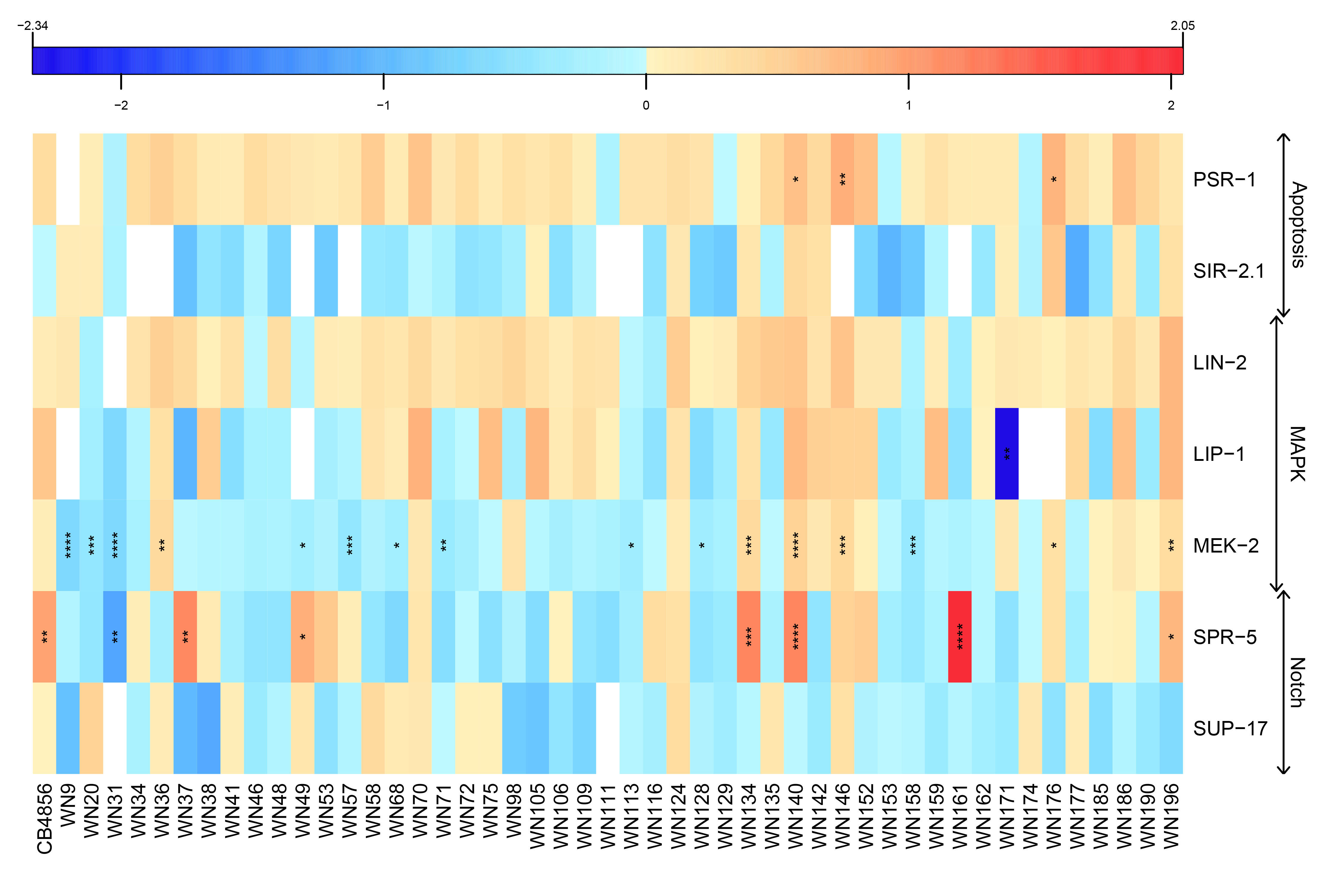

Supplement: S7 Fig — Protein abundance was quantified by SRM. Identification of the true peak group was performed using the mProphet software, followed by protein significance analysis using an intensity-based linear mixed-effects model implemented in MSstats. Number of asterisks represent BH corrected P-values as follows, *P ≤ 0.05; **P ≤ 0.01; ***P ≤ 0.001; ****P ≤ 0.0001. Blue and red shades within heat map represent log2 scaled fold changes in protein abundance relative to N2 (white boxes = no data). (TIF) [file pone.0149418.s007.tif]
